# Effect of transporter inhibition on the distribution of cefadroxil in rat brain

**DOI:** 10.1186/2045-8118-11-25

**Published:** 2014-11-14

**Authors:** Xiaomei Chen, Irena Loryan, Maryam Payan, Richard F Keep, David E Smith, Margareta Hammarlund-Udenaes

**Affiliations:** Department of Pharmaceutical Sciences, College of Pharmacy, University of Michigan, Ann Arbor, Mi 48109 USA; Department of Pharmaceutical Biosciences, Translational PKPD Research Group, Uppsala University, Box 591, SE-75124 Uppsala, Sweden; Biopharmaceutics and Pharmacokinetic Division, Department of Pharmaceutics, Faculty of Pharmacy, Tehran University of Medical Sciences, Tehran, Iran; Department of Neurosurgery, University of Michigan Health System, Ann Arbor, MI 48109 USA

**Keywords:** Blood–brain barrier, Blood-cerebrospinal fluid barrier, Intracerebral microdialysis, Brain slice, Cefadroxil, Multidrug resistance-associated proteins, Organic anion transporters, Organic anion transporting polypeptides, Peptide transporter 2, Probenecid

## Abstract

**Background:**

Cefadroxil, a cephalosporin antibiotic, is a substrate for several membrane transporters including peptide transporter 2 (PEPT2), organic anion transporters (OATs), multidrug resistance-associated proteins (MRPs), and organic anion transporting polypeptides (OATPs). These transporters are expressed at the blood–brain barrier (BBB), blood-cerebrospinal fluid barrier (BCSFB), and/or brain cells. The effect of these transporters on cefadroxil distribution in brain is unknown, especially in the extracellular and intracellular fluids within brain.

**Methods:**

Intracerebral microdialysis was used to measure unbound concentrations of cefadroxil in rat blood, striatum extracellular fluid (ECF) and lateral ventricle cerebrospinal fluid (CSF). The distribution of cefadroxil in brain was compared in the absence and presence of probenecid, an inhibitor of OATs, MRPs and OATPs, where both drugs were administered intravenously. The effect of PEPT2 inhibition by intracerebroventricular (*icv*) infusion of Ala-Ala, a substrate of PEPT2, on cefadroxil levels in brain was also evaluated. In addition, using an *in vitro* brain slice method, the distribution of cefadroxil in brain intracellular fluid (ICF) was studied in the absence and presence of transport inhibitors (probenecid for OATs, MRPs and OATPs; Ala-Ala and glycylsarcosine for PEPT2).

**Results:**

The ratio of unbound cefadroxil AUC in brain ECF to blood (K_p,uu,ECF_) was ~2.5-fold greater during probenecid treatment. In contrast, the ratio of cefadroxil AUC in CSF to blood (K_p,uu,CSF_) did not change significantly during probenecid infusion. *Icv* infusion of Ala-Ala did not change cefadroxil levels in brain ECF, CSF or blood. In the brain slice study, Ala-Ala and glycylsarcosine decreased the unbound volume of distribution of cefadroxil in brain (V_u,brain_), indicating a reduction in cefadroxil accumulation in brain cells. In contrast, probenecid increased cefadroxil accumulation in brain cells, as indicated by a greater value for V_u,brain_.

**Conclusions:**

Transporters (OATs, MRPs, and perhaps OATPs) that can be inhibited by probenecid play an important role in mediating the brain-to-blood efflux of cefadroxil at the BBB. The uptake of cefadroxil in brain cells involves both the influx transporter PEPT2 and efflux transporters (probenecid-inhibitable). These findings demonstrate that drug-drug interactions via relevant transporters may affect the distribution of cephalosporins in both brain ECF and ICF.

## Background

Cephalosporins, a class of beta-lactam antibiotics, have been widely used for the prophylaxis and treatment of a variety of infections [1]. In addition to their antibacterial activity, the therapeutic effects of different cephalosporins depend on their pharmacokinetics and tissue distribution, which are affected by multiple membrane transporters. Some cephalosporins are substrates of proton-coupled oligopeptide transporters (POTs) [2], organic anion transporters (OATs) [3], organic anion transporting polypeptides (OATPs) [4, 5], and multidrug resistance-associated proteins (MRPs) [6, 7]. These transporters are widely distributed in several tissues including the kidney, liver, intestine, and brain [8], influencing cephalosporin absorption, distribution, and elimination.

Among all tissues, drug delivery to brain is the most challenging because of the blood–brain barrier (BBB), situated at the cerebral endothelium, and blood-CSF barrier (BCSFB) at the choroid plexus epithelium [9]. In addition to tight junctions limiting paracellular diffusion [10, 11], the BBB and BCSFB express many transporters responsible for chemical exchange between brain and blood including efflux transporters important for protecting the brain from waste products and potential toxins [12]. Among the cephalosporin transporters, the MRPs and OATs at the BBB and BCSFB are believed to transport substrates from brain (and CSF) to blood as efflux transporters [13–15]. Peptide transporter 2 (PEPT2, a member of POTs) at the apical side (CSF facing) of the BCSFB is able to transport substrates from the CSF side towards blood [16]. The OATPs are expressed both at the BBB and BCSFB as bidirectional transporters [17, 18]. The above mentioned transporters are also expressed on the cell membrane of brain cells (neurons, astrocytes, and microglia) [13, 14, 18, 19], potentially affecting cephalosporin distribution after their entry into brain. Thus, membrane transporters may influence the brain distribution of cephalosporins and influence their effectiveness for treating central nervous system (CNS) infections.

Cefadroxil is a first-generation cephalosporin and used clinically mainly to treat urinary tract infections [1]. The current study employed cefadroxil as a model drug to examine the potential impact of transporters on the brain distribution of cephalosporins, as it has been reported to be a substrate of POTs, OATs, MRPs, and OATPs [5, 6, 20–22]. In small intestine, PEPT1, a member of POTs, mediates peptide/mimetic uptake at the apical side of enterocytes, leading to a high oral bioavailability [23, 24]. Thus, PEPT1 knockout led to a 23-fold reduction in peak plasma concentrations and a 14-fold decrease in systemic exposure of cefadroxil in mice [24]. Also, MRP3 and MRP4, at the basolateral side of enterocytes, may contribute to the further transport of cefadroxil from enterocyte to blood [22]. The kidney is the main elimination organ for cefadroxil and studies in PEPT2 null mice indicate that this is the principal transporter involved in cefadroxil reabsorption [20]. Moreover, the clearance of cefadroxil is significantly reduced by co-administration of probenecid [20, 25]. Probenecid is widely known as an inhibitor of OATs, which mediates renal secretion at the basolateral membrane of proximal tubule epithelia. However, probenecid can also inhibit the MRPs and OATPs that transport substrates from blood to urine via the kidney [26, 27].

Studies on the distribution of cefadroxil in brain have focused on the function of PEPT2 at the BCSFB and brain cells. PEPT2 in choroid plexus removes cefadroxil from CSF. As a result, the CSF-to-blood concentration ratio of cefadroxil in wild-type mice was markedly lower than that in PEPT2 knockout mice [20, 28]. In addition, cefadroxil inhibited the uptake of PEPT2 substrates in rodent neonatal astrocytes, demonstrating an uptake function of PEPT2 in brain cells [19, 29, 30]. However, there are no studies on the influence of transporters on the distribution of cefadroxil in brain extracellular fluid (ECF). A deeper understanding of the effect of transporters on CNS cephalosporin distribution is helpful for the more efficient use of cephalosporins for treating brain infections like meningitis.

The present study examined the impact of transporters on cefadroxil distribution in brain ECF and CSF using probenecid, an inhibitor of OATs, MRPs and OATPs, as well as Ala-Ala, a substrate of PEPT2. *In vivo* microdialysis was applied to measure cefadroxil concentrations in rat brain ECF, CSF and blood. In addition, an *in vitro* brain slice method was performed to study cefadroxil distribution within the rat brain parenchyma.

## Methods

### Chemicals

Cefadroxil, cefadroxil-D4 (deuterated), probenecid, Ala-Ala, glycylsarcosine (GlySar), and amoxicillin were purchased from Sigma-Aldrich (St. Louis, MO, USA), isoflurane from Baxter Medical AB (Kista, Sweden), and 100 IU/mL heparin from Leo Pharma AB (Malmö, Sweden). Acetonitrile and formic acid were obtained from Merck (Darmstadt, Germany). All other chemicals were of analytical grade or better. Ringer’s solution was used to perfuse the microdialysis probes and consisted of 145 mM NaCl, 0.6 mM KCl, 1.0 mM MgCl_2_, and 1.2 mM CaCl_2_ in 2 mM phosphate buffer, pH 7.4. Artificial extracellular fluid (aECF), used to buffer the brain slices, was comprised of 10 mM glucose, 129 mM NaCl, 3 mM KCl, 1.2 mM MgSO_4_, 0.4 mM K_2_HPO_4_, 1.4 mM CaCl_2_, and 25 mM HEPES, pH 7.6, at room temperature. Normal saline was obtained from Braun Medical AB (Stockholm, Sweden) and the Milli-Q system (Millipore, Bedford, Massachusetts) was used to purify the water.

### Animals

Male Sprague–Dawley rats (260–300 g) were obtained from Taconic (Lille Skensved, Denmark). Rats were acclimated for at least 7 days in a temperature- and humidity-controlled environment with 12-hour light/dark cycles before study. The protocols in this study were approved by the Animal Ethics Committee of Uppsala University, Sweden (C351/11 and C328/10).

### Microdialysis study of cefadroxil in the absence and presence of probenecid

Surgery was performed one day before microdialysis in order to implant vessel catheters and microdialysis probes as described previously [31] with modification. Briefly, under isoflurane anesthesia and with body temperature controlled at 38°C (CMA/150 temperature controller, CMA, Stockholm, Sweden), catheters were inserted into the left femoral vein for cefadroxil infusion, the left jugular vein for control (Day 1) or probenecid infusion (Day 2), and the left femoral artery for blood sampling. A CMA/20 probe with 10 mm polyarylethersulphone (PAES) membrane was inserted into the right jugular vein. The rat was then fixed on a stereotaxic frame equipped with an anesthesia mask. Two guide cannulas were implanted into the brain striatum (ST coordinates, +0.2 mm anteroposterior, −4.7 mm lateral, −3.8 mm dorsoventral with an angle of 15° at the coronal plane towards midline) and lateral ventricle (LV coordinates, −0.9 mm anteroposterior, +1.6 mm lateral, −2.9 mm dorsoventral), and fixed to the skull by a screw and dental cement. A CMA 12 probe with 3 mm PAES membrane was inserted into the striatal guide cannula for monitoring brain ECF and a CMA 12 probe with 1 mm PAES membrane was inserted into the ventricular guide cannula for CSF sampling. At the end of the surgery, the rat was placed in a CMA 120 system for freely moving animals in which it had free access to food and water, and allowed to recover for 24 hours before experimentation.

On Day 1, a 90-min stabilization period was performed in which Ringer’s solution, containing cefadroxil-D4, was perfused through the microdialysis probes by pump (CMA 400, Solna, Sweden) at a flow rate of 0.5 μL/min. During this period, and throughout the entire experiment (another 420 min), microdialysis samples (10 μL each) were collected every 20 min using a fraction collector (CMA 142, Solna, Sweden) and stored at 4°C until analysis. To quantify unbound drug concentrations in brain and blood, cefadroxil-D4 was used to calibrate the probes using retrodialysis [32]. Because cefadroxil levels in brain and blood were quite different, 1 μg/mL cefadroxil-D4 was used to perfuse the blood probe and 0.1 μg/mL for the brain probe. At 90 min, cefadroxil solution (6 mg/mL in normal saline) was administered intravenously (*iv*) as a bolus infusion of 0.3 mg/kg/min for 20 min followed by a constant-rate infusion of 0.15 mg/kg/min for 160 min (for a total of 180 min). In addition to the microdialysis samples, arterial blood samples (100 μL) were drawn predose and at 5, 18, 90, 150, 185, 190, 210, 240, 300, and 420 min after initiating the cefadroxil bolus infusion. Plasma was harvested from blood after centrifuging at 7200 g for 5 min and then frozen at −20°C until analysis. On Day 2, the cefadroxil experiment was repeated, however, 15 mg/mL probenecid in 5% NaHCO_3_ in saline (as opposed to 5% NaHCO_3_ in saline only on Day 1) was added as a 20 mg/kg bolus followed by 20 mg/kg/hr infusion for 420 min (i.e., cefadroxil in the presence of probenecid).

### Microdialysis study of cefadroxil in the absence and presence of Ala-Ala

The surgery and microdialysis method for this study was similar to that described before for probenecid except, in this case, the dipeptide Ala-Ala was administered instead and by intracerebroventricular (*icv*) infusion. In order to perform the microdialysis sampling and *icv* infusion simultaneously, a microdialysis probe with an additional infusion cannula passing through the lumen of probe (IBR combination probe with 1 mm polyacrylanitrile membrane, BASi, West Lafayette, IN, USA) was implanted into the lateral ventricle (coordinates, −0.9 mm anteroposterior, −1.6 mm lateral, −2.9 mm dorsoventral). For these studies (i.e., cefadroxil in the absence and presence of Ala-Ala), the experiment was performed in one day. In brief, following the 90-min stabilization period, cefadroxil saline solution was infused *iv* at 0.3 mg/kg/min for 20 min followed by 0.15 mg/kg/min for 400 min (for a total of 420 min). An *icv* infusion of Ringer’s solution, 0.3 μL/min, was started 30 min prior to cefadroxil administration and maintained for another 240 min (control phase). At this time, an *icv* infusion of 0.32 mg/mL Ala-Ala in Ringer’s solution was started and then maintained for another 180 min.

### *In vitro*brain slice study

The brain slice protocol was based on a previously published method with minor modifications [33]. Briefly, fresh brains were collected in which six 300-μm coronal slices were prepared from each animal using a microtome (Leica VT1200, Leica Microsystems AB, Sweden). Resultant slices were transferred to an 80-mm diameter beaker with 15 mL aECF containing 0.8 μM cefadroxil with or without 5 mM GlySar, 5 mM Ala-Ala, or 1 mM probenecid. Covered by a lid comprised of a Teflon fluorinated ethylene-propylene film (DuPont, Katco Ltd, UK), the beaker was incubated in a shaker (MaxQ4450, Thermo Fisher Scientific, Nino Lab, Sweden) at 45 rpm, 37°C, for 2 hr. Throughout the incubation, there was a constant flow of oxygen into the shaking chamber to maintain slice viability. After incubation, 200 μL of blank rat brain homogenate without cefadroxil was added to 200 μL of buffer sample to keep the matrix consistent among all the samples for the following analysis. The brain slices were then weighed, after drying on filter paper, and homogenized individually in aECF (9:1 ratio, w/v) using an ultrasonic processor (VCX-130, Sonics, Chemical Instruments AB, Sweden). All samples were stored at −20°C until analysis.

In all experiments, coronal slices were prepared from the same anatomical plane corresponding to the striatal region (no midbrain structures) in order to avoid potential discrepancies in the assessment of the unbound volume of distribution of cefadroxil in brain (Vu,brain). In our studies, the Vu,brain values of cefadroxil were similar in each rat with little variability (mean coefficient of variation ≤5.4%). Potential regional differences in the Vu,brain of cefadroxil were not studied.

### Chemical analysis

The analysis of cefadroxil (and cefadroxil D-4) was carried out using liquid chromatography–tandem mass spectrometry (LC-MS/MS). Specifically, 5 μL microdialysis samples were injected into the LC-MS/MS after adding amoxicillin solution as an internal standard. For plasma and homogenate samples, the proteins were precipitated by adding acetonitrile at a ratio of 1:3. After centrifuging at 7200 g for 3 min, the supernatant was diluted with 0.1% formic acid before injecting into the LC-MS/MS. Standard curves and quality control samples were used to quantify and validate the concentrations of cefadroxil in all biological matrices from the study.

Chromatographic separation was achieved on a HyPurity C18 column (50 × 4.6 mm, particle size 3 μm) protected by a HyPurity C18 guard-column (10 × 4.0 mm, particle size 3 μm; Thermo Hypersil-Keystone, PA, USA). A gradient elution involving mobile phase A (0.1% formic acid) and mobile phase B (0.1% formic acid in 1:1 acetonitrile:water) was delivered by two Shimadzu LC-10ADvp pumps (Shimadzu, Kyoto, Japan) at 0.8 mL/min, which was split to 0.3 mL/min before entering the MS detector. A Quattro Ultima Pt mass spectrometer (Waters, Milford, MA, USA) was used for detection on positive electrospray ionization (ESI+) mode. The transition mode was m/z 363.9 → 207.9 for cefadroxil, m/z 368.0 → 212.0 for cefadroxil-D4, and m/z 366.0 → 348.9 for amoxicillin. All data were acquired and processed using Masslynx 4.1 (Waters, Milford, MA, USA).

### Data analysis

The relative recovery of cefadroxil in each microdialysis probe was estimated from retrodialysis of the calibrator, cefadroxil-D4, and calculated as:
1

where *C*_*in,CEF-D4*_ is the concentration of cefadroxil-D4 in perfusate and *C*_*out,CEF-D4*_ is the concentration of cefadroxil-D4 in dialysate. The unbound concentrations of cefadroxil in blood (*C*_*u,blood*_), brain ECF (*C*_*u,ECF*_), and CSF (*C*_*u,CSF*_) were calculated from their respective concentrations in dialysate (*C*_*dialysate*_) as:
2

For the microdialysis study of cefadroxil (with and without probenecid), the trapezoidal method was used to calculate area under the curve for unbound cefadroxil (AUC_u_) in blood, ECF, and CSF from 0–420 min. AUC_u_ values from 420 min to infinity were determined by extrapolation from the time of the last measured concentration *C*_*last*_ according to , in which λ_z_ is the terminal rate constant obtained from the slope of the last 7 observations. The blood concentration of cefadroxil at steady-state (C_u,ss,blood_) was calculated from the average of concentrations during the 120–180 min time period. The unbound partition coefficient of cefadroxil in brain ECF (*K*_*p,uu,ECF*_) and CSF (*K*_*p,uu,CSF*_) was obtained as follows:
34

Non-compartmental analyses were performed using the microdialysis samples from blood to obtain the pharmacokinetic parameters of unbound cefadroxil, in which area under the moment curve (AUMC_u_) was also obtained by trapezoidal method. The mean input time (MIT) was 66 min calculated from , where R0 and Tin denote the infusion rate and infusion time of the two consecutive cefadroxil infusions. With the correction of MIT, the mean residence time with an *iv* bolus (MRT_iv_) was obtained:
5

The total clearance (CL), volume of distribution steady-state (V_ss_), and half-life (t_1/2_) were calculated based on the total cefadroxil dose (30 mg/kg, which includes both the bolus and constant-rate infusions), along with AUC_u_ and AUMC_u_ from times zero to infinity (inf):
678

For the microdialysis study of cefadroxil with and without Ala-Ala, K_p,uu_ was calculated from the unbound concentration of drug at steady-state (C_u,ss,ECF_ or C_u,ss,CSF_) by:
9

where *C*_*u,ss*_ was calculated during the 120–200 min time period for the control phase (i.e., without Ala-Ala) and during the 320–420 min time period for the dipeptide phase (i.e., with Ala-Ala).

In analyzing brain slice data, the unbound volume of distribution in brain (*V*_*u,brain*_, in mL/g brain) was calculated for cefadroxil as:
10

where *A*_*brain*_ is the total amount of cefadroxil in brain slice, *C*_*buffer*_ is the concentration of cefadroxil in buffer at the end of incubation, and *V*_*i*_ is the volume of buffer film surrounding the brain slice because of incomplete adsorption by the filter paper; *V*_*i*_ was reported as 0.094 mL/g brain [34].

### Statistical analysis

Data are expressed as mean ± SEM. A two-tailed paired t-test was used to compare cefadroxil parameters between the control and inhibition phases. A value of *p* <0.05 was considered statistically significant. For the brain slice study, a one-way ANOVA with Dunnett’s test was performed to compare each treatment group to the control. GraphPad Prism v5.04 (GraphPad Software Inc., San Diego, CA) was used for all statistical analyses.

## Results

### Microdialysis study of cefadroxil in the absence and presence of probenecid

**Figure 1:**
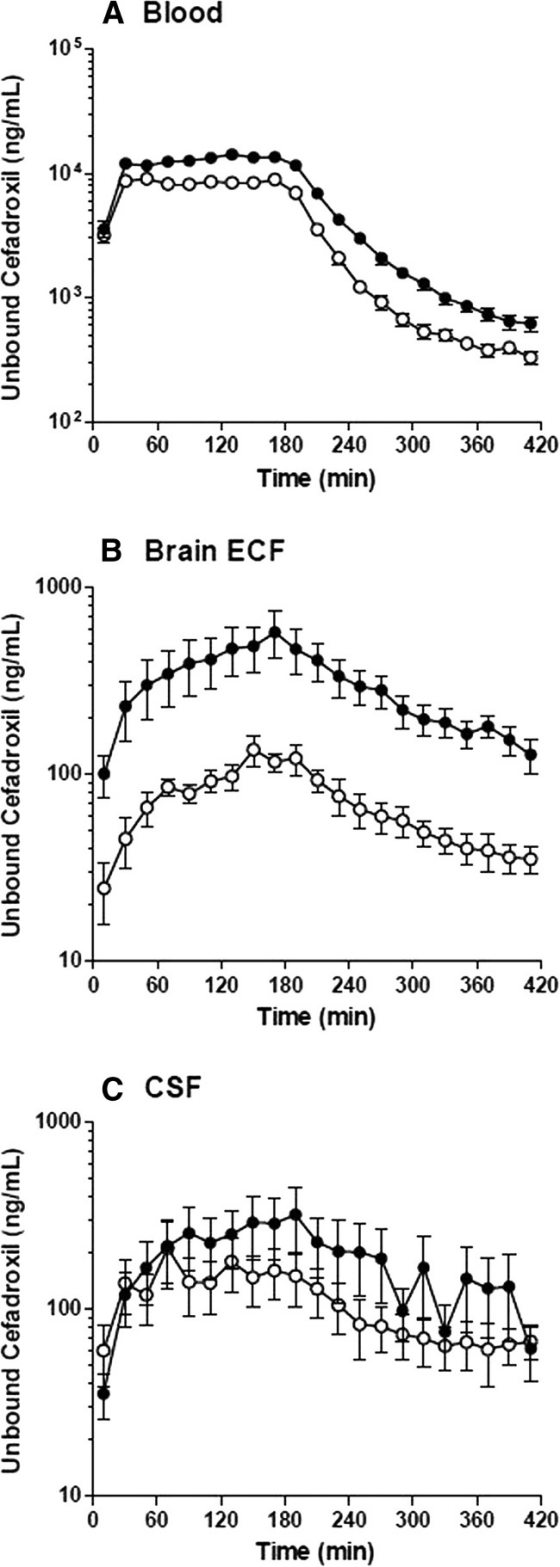
**The concentration-time profiles of unbound cefadroxil in rat blood (A), brain ECF (B), and CSF (C) in the absence and presence of probenecid.** Open circles represent the results from Day 1 (no probenecid) and solid circles the results from Day 2 (with probenecid). Data are expressed as mean ± SEM (n = 6).

There were no significant differences in probe relative recoveries between the two days. The recoveries were 14 ± 1% for the 3-mm probe in brain ECF, 6.7 ± 1.1% for the 1-mm probe in lateral ventricle, and 71 ± 2% for the 10-mm probe in blood. As shown in Figure 1A, steady-state concentrations of cefadroxil in blood were quickly achieved after the bolus infusion of 0.3 mg/kg/min for 20 min followed by the constant-rate infusion of 0.15 mg/kg/min for 160 min. Compared to Day 1 (control phase), probenecid infusion increased C_u,ss,blood_ and AUC_u_ of cefadroxil by ~60%. The elevated systemic exposure probably resulted from a decrease in cefadroxil clearance from 16.9 ± 1.0 to 10.7 ± 0.7 mL/min/kg (Table 1). However, the MRT and t_1/2_ did not differ significantly between days, reflecting a reduced volume of distribution (V_ss_) with probenecid, indicating probenecid may decrease the accumulation of cefadroxil in certain tissues. Plasma cefadroxil concentrations (data not shown) were comparable to the unbound blood concentrations from microdialysis, consistent with previous studies showing that the unbound fraction of cefadroxil in plasma (*fu*) is nearly 1.0 [35].

**Table 1:** **Pharmacokinetic parameters of unbound cefadroxil in rat blood and brain on Day 1 (Control, Ctrl) and Day 2 (with probenecid, Pro)**

Parameters	Unit	Day 1 (Ctrl)	Day 2 (Pro)	Pro/Ctrl
**Blood**				
AUC_u_ (0–420)	μg*min/mL	1747 ± 90	2801 ± 175***	1.60
AUC_u_ (0-inf)	μg*min/mL	1802 ± 97	2873 ± 177***	1.59
C_u,ss,blood_	μg/mL	8.5 ± 0.4	13.8 ± 0.9***	1.62
MRT_iv_	min	71 ± 4	77 ± 4	1.05
t_1/2_	min	49 ± 2	53 ± 3	1.09
CL	mL/min/kg	16.9 ± 1.0	10.7 ± 0.7***	0.63
V_ss_	L/kg	1.19 ± 0.12	0.82 ± 0.10***	0.69
**Brain ECF**				
AUC_u_ (0–420)	μg*min/mL	31 ± 5	122 ± 31*	3.93
AUC_u_ (0-inf)	μg*min/mL	40 ± 7	174 ± 35**	4.37
K_p,uu,ECF_ (0–420)		0.018 ± 0.003	0.042 ± 0.009*	2.35
K_p,uu,ECF_ (0-inf)		0.022 ± 0.003	0.058 ± 0.009*	2.63
**Brain CSF**				
AUC_u_ (0–420)	μg*min/mL	39 ± 12	73 ± 27	1.88
AUC_u_ (0-inf)	μg*min/mL	57 ± 15	117 ± 50	2.04
K_p,uu,CSF_ (0–420)		0.022 ± 0.006	0.024 ± 0.008	1.13
K_p,uu,CSF_ (0-inf)		0.031 ± 0.007	0.039 ± 0.015	1.26

Data are expressed as mean ± SEM (n = 6). A paired t-test was performed to compare cefadroxil parameters between the control (without probenecid) and treatment (with probenecid) phases of the study. *p < 0.05, **p < 0.01, and ***p < 0.001. Abbreviations: *AUC*_*u*_, Area under the unbound concentration-time curve from time zero to 420 min (0–420) or from time zero to infinity (0-inf); *C*_*u,ss,blood*_, Unbound steady-state blood concentration; *MRT*_*iv*_, Mean residence time; t_1/2_, Half-life; *CL*, Total clearance; *V*_*ss*_, Volume of distribution steady-state; *K*_*p,uu,ECF*_, Ratio of AUC_u_ in brain ECF to AUC_u_ in blood; and *K*_*p,uu,CSF*_, Ratio of AUC_u_ in CSF to AUC_u_ in blood.

**Figure 2:**
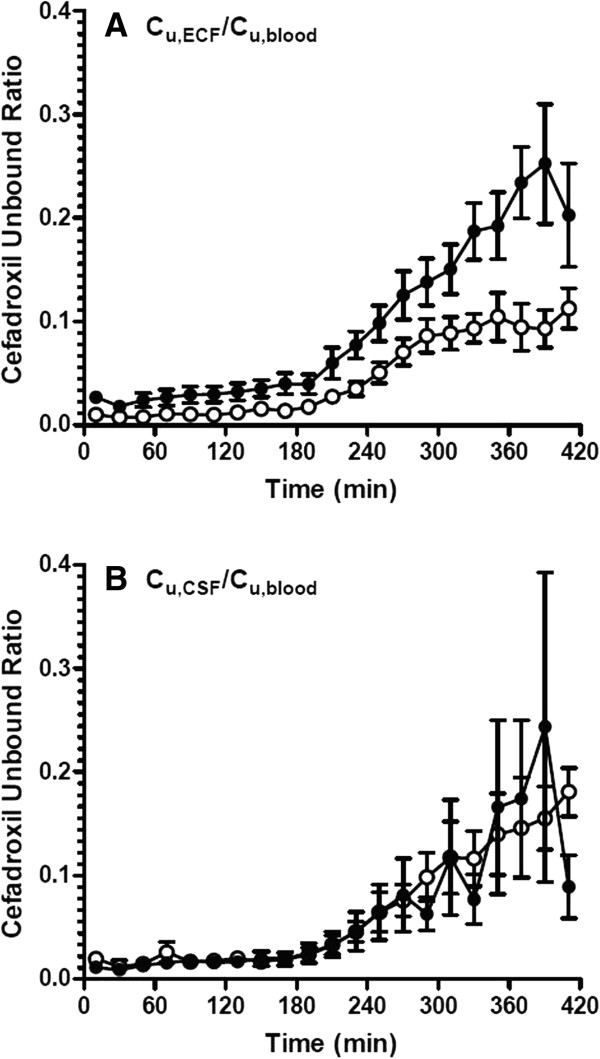
**The ratio of unbound cefadroxil in rat brain ECF (A) or CSF (B) to that in blood versus time.** Open circles represent the results from Day 1 (no probenecid) and solid circles the results from Day 2 (with probenecid). Data are expressed as mean ± SEM (n = 6).

**Figure 3:**
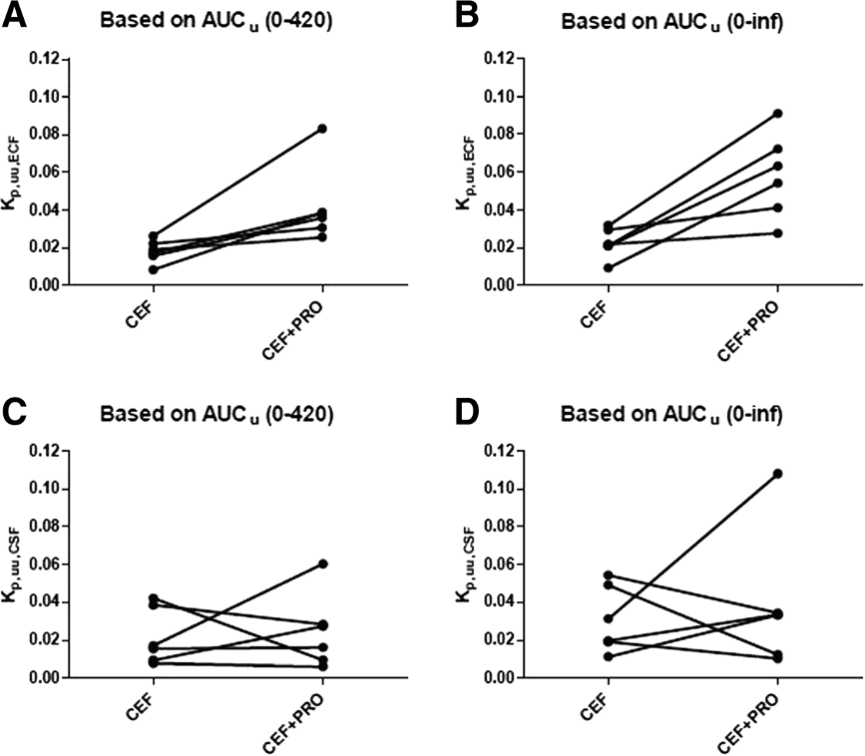
**The unbound partition coefficient (K**_**p,uu**_ **) of cefadroxil in rat brain ECF (A, B) and CSF (C, D) for each of the six animals.** CEF represents the study in which cefadroxil is given alone (Day 1) and CEF + PRO is when cefadroxil is given in the presence of probenecid (Day 2). See Table 1 for statistical analyses.

In addition to increasing unbound cefadroxil blood concentrations, probenecid increased the AUC_u_ of drug in brain ECF 4-fold (*p* <0.05) and the AUC_u_ of drug in CSF 2-fold (*p* >0.05) (Figures 1B and 1C, and Table 1). To determine if cefadroxil penetration into brain was affected by probenecid, brain drug concentrations were corrected by the corresponding values in blood (Figure 2). During probenecid infusion (Day 2), the C_u,ECF_ values of cefadroxil, relative to blood, were higher than control (Day 1) at all time points. In contrast, the C_u,CSF_ values of cefadroxil, relative to blood, were comparable. To evaluate the effect of probenecid on cefadroxil penetration into brain, the unbound partition coefficient K_p,uu_ was calculated for both brain ECF and CSF using AUC_u_ values from 0–420 min and from 0-infinity (Figure 3). K_p,uu_ was around 0.02 in both brain ECF and CSF in the control situation, indicating limited penetration of cefadroxil into brain and extensive efflux at the BBB (Table 1). K_p,uu,ECF_ values were about 2.5-fold greater with probenecid treatment as compared to control. In contrast, there were no significant differences in K_p,uu,CSF_ between control and probenecid treatments. This may reflect, in part, greater experimental variability in the direction of change for this parameter (Figures 3C and 3D).

### Microdialysis study of cefadroxil in the absence and presence of Ala-Ala

**Figure 4:**
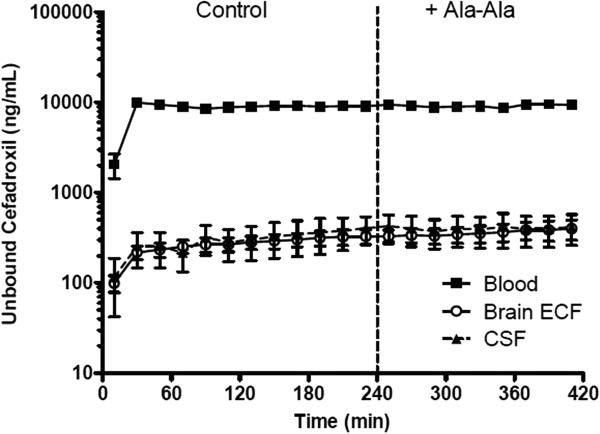
**The concentration-time profiles of unbound cefadroxil in rat blood, brain ECF, and CSF in the absence and presence of Ala-Ala.** Solid squares represent the results in blood, open circles the results in brain ECF, and solid triangles the results in CSF. The vertical dashed line separates the two treatment phases (CEF ± Ala-Ala). Data are expressed as mean ± SEM (n = 7).

Recoveries were 16 ± 2%, 12 ± 1%, and 72 ± 1% for probes in the striatum, lateral ventricle and blood, respectively. Ala-Ala is a natural dipeptide that can be degraded in the body; thus, Ala-Ala was infused by the *icv* route in order to achieve high concentrations in CSF. The goal of the study was to determine if Ala-Ala affects the distribution of cefadroxil by comparing levels in ECF and CSF between vehicle control phase and during Ala-Ala infusions. As shown in Figure 4, the unbound concentrations of cefadroxil did not change substantially in brain ECF or CSF during Ala-Ala infusions. Furthermore, there was no significant difference between control and Ala-Ala infusions in K_p,uu,ECF_ (0.033 ± 0.004 to 0.041 ± 0.008, *p* = 0.15) or K_p,uu,CSF_ (0.038 ± 0.017 to 0.043 ± 0.016, *p* = 0.43).

### *In vitro*brain slice study

**Figure 5:**
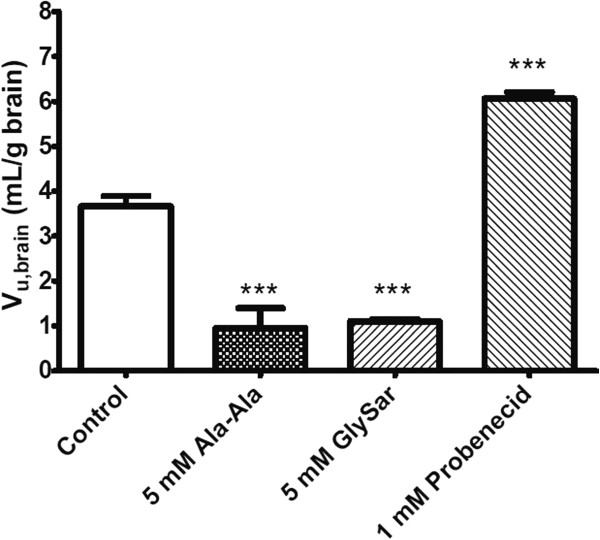
**The unbound volume of distribution of cefadroxil (V**_**u,brain**_ **) in rat brain slices.** Studies were performed with 0.8 μM cefadroxil alone (Control) and in the presence of inhibitors (Ala-Ala, GlySar and Probenecid treatments). Data are expressed as mean ± SEM (n = 3-4). One-way ANOVA followed by the Dunnett’s test was performed to compare the inhibitor and control phases. ***p < 0.001 compared to control.

V_u,brain_ describes the relationship between the total amount of drug in brain and the unbound concentration of drug in ECF, and is useful as a measure of intra-parenchymal distribution [36]. A higher value V_u,brain_ suggests that more drug accumulates inside the brain cells. For control brain slices, the V_u,brain_ of cefadroxil was 3.67 ± 0.23 mL/g brain (Figure 5). Two PEPT2 substrates, Ala-Ala and GlySar, reduced the V_u,brain_ of cefadroxil to 0.95 ± 0.45 and 1.10 ± 0.05 mL/g brain, respectively, indicating that they reduced the accumulation of cefadroxil inside brain cells (*p* < 0.001). In contrast, probenecid increased the V_u,brain_ of cefadroxil to 6.06 ± 0.15 mL/g brain, suggesting that probenecid led to more accumulation of cefadroxil inside brain cells (*p* < 0.001).

## Discussion

The current study used microdialysis and brain slice methods to examine the transport mechanisms affecting the distribution of cefadroxil, a cephalosporin antibiotic, in brain. The results demonstrated that: 1) co-administration of probenecid increased blood cefadroxil levels; 2) probenecid markedly increased brain ECF cefadroxil concentrations; 3) the probenecid effect on brain ECF levels were partially due to increased blood concentrations but also due to inhibition of cefadroxil efflux at the BBB (OATs, OATPs and/or MRPs); 4) in contrast, increased CSF cefadroxil concentrations with probenecid were only due to elevated blood concentrations of antibiotic; 5) intracerebroventricular infusion of the PEPT2 substrate, Ala-Ala, did not increase brain ECF or CSF cefadroxil levels; and 6) brain slice experiments demonstrated that PEPT2 was involved in the uptake of cefadroxil into brain cells and that probenecid blocked a mechanism transporting cefadroxil out of cells.

**Figure 6:**
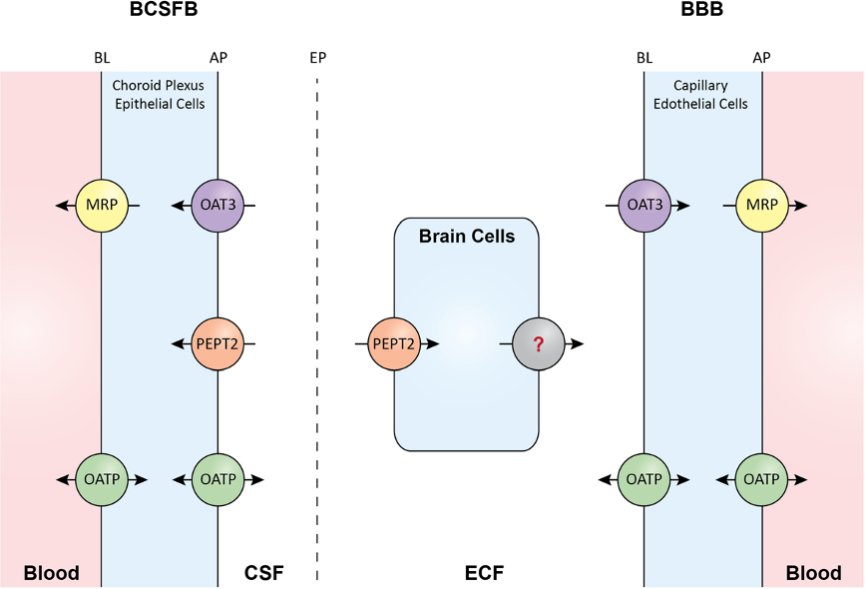
**Membrane transporters (potentially) involved in the CNS distribution of cefadroxil.** Several references were used to inform this schematic representation [16, 39–41]. There is much debate regarding the isoforms and membrane localization of MRPs at the BBB. There is, though, considerable evidence for some MRPs having an apical distribution clearing substrates to blood as depicted. There is also functional evidence for the probenecid-inhibitable efflux of cefadroxil from brain cells , the nature of which is uncertain but may include OAT, MRP and/or OATP transporters. BL represents the basolateral membrane, AP the apical membrane, and EP the ependyma.

In the interaction study between cefadroxil and probenecid, intravenous co-administration of probenecid reduced the clearance of cefadroxil. This finding was consistent with previous studies [20, 25] showing that probenecid inhibits the renal secretion of many cephalosporins by OATs (and perhaps MRPs and OATPs) at the kidney proximal tubule [37]. Even though steady-state concentrations were achieved quickly for unbound cefadroxil in blood, steady-state concentrations in brain ECF were not fully reached within the infusion period of 3 hr. As a consequence, C_u,ECF_ decreased more slowly than C_u,blood_ after termination of the cefadroxil infusion. The above phenomenon may be due to low permeability of passive diffusion of cefadroxil at the BBB, considering its high hydrophilicity. The K_p,uu_ of brain ECF is determined by the net influx and efflux clearances at the BBB, as K_p,uu_ = CL_in_/CL_out_ [36]. If only passive transport occurs at the BBB, K_p,uu_ is equal to unity due to the equal values for CL_in_ and CL_out_. However, the K_p,uu,ECF_ of cefadroxil was about 0.02, indicating that cefadroxil CL_out_ is much higher than CL_in_. Thus, it appears that there is net efflux transport for cefadroxil at the BBB. It has been reported that cefadroxil is a substrate of OATs and MRPs [6, 21, 22]. Specifically, OAT3 located at the basolateral (abluminal) side of the BBB and MRPs at the apical (luminal) side of the BBB mediate brain-to-blood transport as efflux transporters, thus possibly contributing the low K_p,uu,ECF_ of cefadroxil [13, 15, 38]. Inhibition of OAT3 and/or MRPs at the BBB is the probable reason why probenecid increased the K_p,uu,ECF_ of cefadroxil ~2.5 fold. In addition to OATs and MRPs, cefadroxil was reported to be a substrate of OATPs. However, OATPs are bidirectional transporters [12, 17, 18] and their net effect on cefadroxil transport at the BBB is unknown. A schematic representation of the membrane transporters involved in the CNS distribution of cefadroxil is shown in Figure 6.

OATs and MRPs [13, 14] are also responsible for the transport of substrates from CSF to blood at the BCSFB. Therefore, it was expected that inhibition of OATs and MRPs by probenecid would increase the K_p,uu,CSF_ of cefadroxil. However, no significant change was found for this parameter. The differential effect of transporter inhibition by probenecid on the distribution of cefadroxil in brain ECF and CSF may be related to the physiological and structural differences between BBB and BCSFB. The complement of efflux transporters, their expression levels, and cellular location may affect the relative importance of individual transporters in each of the two systems. In addition, the endothelial BBB is tighter than the epithelial BCSFB (choroid plexus), affecting paracellular diffusion [42]. A recent study on the effects of probenecid on methotrexate transport found a different modulation of methotrexate distribution in brain ECF and CSF [43]. There was a dose-dependent effect, in which probenecid increased the brain ECF-to-plasma ratio for two dose regimens of methotrexate (40 mg/kg and 80 mg/kg), whereas probenecid only significantly increased the CSF-to-plasma ratio at the higher dose [43]. The differential effects of probenecid on cefadroxil at the BBB and BCSFB in our study are unlikely to be due to differences in inhibitor concentration at the two sites as Deguchi *et al.* [44] found higher probenecid concentrations in CSF than ECF after systemic dosing.

In a previous study, PEPT2 ablation resulted in a marked increase in the CSF-to-blood concentration ratio of cefadroxil, indicating the importance of PEPT2 in eliminating cefadroxil from CSF at the BCSFB [20]. However, in the present study, an *icv* infusion of the PEPT2 substrate Ala-Ala did not significantly change CSF cefadroxil concentrations. This lack of effect may reflect insufficient concentrations of Ala-Ala reaching the BCSFB. Ala-Ala was chosen to inhibit PEPT2 because it has a relatively high affinity for that transporter (K_i_ = 6.3 μM, similar to that of cefadroxil with a K_i_ = 3.0 μM) [2]. However, Ala-Ala has the disadvantage of being degraded by peptidases, many of which are found in the choroid plexus and brain [45].

V_u,brain_ is a measure of drug distribution within brain parenchyma. The water volume in brain parenchyma is 0.8 mL/g brain and a V_u,brain_ of around 0.8 mL/g brain indicates a drug is distributed evenly through the whole brain tissue [36]. From a previous study using equilibrium dialysis in brain homogenate (data not published), cefadroxil had a fraction unbound (*fu*) of nearly 1, indicating little, if any, drug binding to brain tissues. This, together with the cefadroxil V_u,brain_ of 3.67 mL/g brain in the present study indicates the presence of uptake transporter(s) at the membrane of brain cells. The PEPT2 substrates, 5 mM Ala-Ala and GlySar, reduced the V_u,brain_ of cefadroxil, indicating that competitive inhibition of PEPT2 decreased the uptake of cefadroxil into brain cells. This is consistent with previous findings that PEPT2 is expressed on neurons and responsible for cellular uptake [46]. In contrast, probenecid increased the V_u,brain_ of cefadroxil, indicating there may also be efflux transporters (e.g., OATs, MRPs or OATPs) removing cefadroxil from brain cells. Interestingly, a previous study demonstrated that probenecid increased the intracellular levels of valproic acid by 1.5-fold in rabbit brain during *in vivo* microdialysis [47].

By using intracerebral microdialysis *in vivo* and brain slices *in vitro*, a better understanding was obtained about the effect of transporters on cefadroxil distribution in brain and, specifically, in brain extracellular and intracellular fluids, and CSF. From our study, it appears that transporters which are probenecid inhibitable (i.e., OATs, MRPs and/or OATPs) move cefadroxil in a vectorial direction from brain ECF to blood, and that PEPT2 transports cefadroxil into brain cells. In addition, as probenecid increased cefadroxil uptake into brain slices, there is an as yet unidentified cefadroxil transporter effluxing this cephalosporin from brain cells. It is concluded that multiple transporters play a role in the distribution of cefadroxil into and within the brain. The impact of these transporters on specific cephalosporins will depend on transporter affinities and drug levels in brain. Microdialysis is a useful tool to study the kinetics of unbound drug concentrations in ECF and CSF [48]. The brain slice method, together with other tools like equilibrium dialysis, provides an approach to study the distribution of drugs within brain after passing the BBB and BCSFB [34, 49].

A deeper understanding of the brain distribution of cephalosporins may aid in the better use of these antibacterial agents for the prophylaxis and treatment of CNS infections. Bacterial meningitis is an inflammatory process of the leptomeninges caused by bacterial infections. Bacterial meningitis is the most frequent CNS infection with a mortality rate approaching 20% [50]. It is believed that bacteria enter the CNS across BBB or BCSFB via transcytosis and finally enter the CSF [50]. Even though BBB permeability increases during meningitis [51], the barriers and their efflux transporters still play a role in limiting cephalosporin entry to brain. Clinically, the cephalosporins used for meningitis are limited to ceftriaxone, cefotaxime, ceftazidime, and cefepime, which have high penetration into CSF [52]. Another CNS infection is cerebritis, a focal brain parenchyma infection, which is often followed by brain abscesses and permanent damage [53]. Treatment for cerebritis and brain abscesses also involves antibiotics. The strategy of blocking the related efflux transporters at the BBB and BCSFB is a promising way to enhance the penetration of relevant cephalosporins into brain ECF and CSF.

Probenecid was firstly widely used to decrease renal clearance of penicillin during World War II, when antibiotic supplies were low. Probenecid decreases the elimination rate and volume of distribution for a variety of medications including most cephalosporins [54]. However, with easier and cheaper production of antibiotics, probenecid is now seldom used with antibiotics. The present study showed that probenecid was able to increase the distribution of cefadroxil in brain ECF not only by reducing the renal clearance (and increasing systemic exposure) but also by specifically increasing the penetration into brain (i.e., increased K_p,uu_) and further into brain cells. It should be appreciated that, although this study was not designed to study cefadroxil under clinical dosing conditions, the co-administration of probenecid allowed cefadroxil to reach the lower limit of its minimal inhibitory concentration in brain ECF for some bacteria (i.e., about 0.4 μg/mL). Thus, the combined therapy of cefadroxil (or perhaps other cephalosporins) and probenecid might be useful for some cases of meningitis and brain abscesses. Whether or not this approach is feasible would depend upon the extent of this drug-drug interaction in patients during different dosing combinations of both antibiotic and the inhibitor. Moreover, there is a delicate balance between the dose–response relationships of bacterial kill and CNS toxicity, which of course would have to be taken into account.

## Conclusions

Using *in vivo* microdialysis and *in vitro* brain slice methods in rat, the present study demonstrated that probenecid increased cefadroxil distribution into brain extracellular and intracellular fluids by blocking related efflux transporters at the BBB and brain cells. Our findings suggest that the combination of probenecid and some cephalosporins may provide a strategy to increase therapeutic drug levels in brain for better treatment of CNS infections like bacterial meningitis and brain abscesses. On the other hand, since multiple transporters are involved in transporting cephalosporins in brain, there is also the potential for drug-drug interactions to enhance cephalosporin-induced neurotoxicity.

## Abbreviations

aECF: Artificial extracellular fluid
CSF: Cerebrospinal fluid
BBB: Blood–brain barrier
BCSFB: Blood-cerebrospinal fluid barrier
CNS: Central nervous system
POT: Proton-coupled oligopeptide transporter
OAT: Organic anion transporter
OATP: Organic anion transporting polypeptide
MRP: Multidrug resistance-associated protein
PEPT2: Peptide transporter 2
CEF: Cefadroxil
PRO: Probenecid
GlySar: Glycylsarcosine
AUC: Area under the curve
t_1/2_: Half-life
inf: Infinity
CL: Clearance
MRT: Mean residence time
AUMC: Area under the moment curve
V_ss_: Volume of distribution steady-state
